# Effect of PCL/nHAEA nanocomposite to osteo/odontogenic differentiation of dental pulp stem cells

**DOI:** 10.1186/s12903-022-02527-1

**Published:** 2022-11-16

**Authors:** Ehsaneh Azaryan, Mohammad Yahya Hanafi-Bojd, Esmat Alemzadeh, Fariba Emadian Razavi, Mohsen Naseri

**Affiliations:** 1 Student Research Committee, Birjand University of Medical Sciences, Birjand, Iran; 2 Cellular and Molecular Research Center, Department of Molecular Medicine, Birjand University of Medical Sciences, Birjand, Iran; 3 Cellular and Molecular Research Center, Birjand University of Medical Sciences, Birjand, Iran; 4 Department of Pharmaceutics and Pharmaceutical Nanotechnology, School of Pharmacy, Birjand University of Medical Siences, Birjand, Iran; 5 Infectious Diseases Research Center, Birjand University of Medical Sciences, Birjand, Iran; 6 Department of Medical Biotechnology, Faculty of Medicine, Birjand University of Medical Science, Birjand, Iran; 7 Dental Research Center, Faculty of Dentistry, Birjand University of Medical Sciences, Birjand, Iran

**Keywords:** Tissue engineering, Hydroxyapatite, Stem cell, *Elaeagnus angustifolia*, Dentin regeneration

## Abstract

**Purpose:**

The green synthesis of nanoparticles has recently opened up a new route in material production. The aim of this study was to evaluate the effect of nanohydroxyapatite (nHA) synthesized from *Elaeagnus angustifolia* (EA) extract in polycaprolactone (PCL) nanofibers (PCL/nHAEA) to odontogenic differentiation of dental pulp stem cells (DPSCs) and their potential applications for dentin tissue engineering.

**Methods:**

Green synthesis of nHA via EA extract (nHAEA) was done by the sol–gel technique. Then electrospun nanocomposites containing of PCL blended with nHA (P/nHA) and nHAEA (P/nHAEA) were fabricated, and the characterization was evaluated via X-ray diffraction (XRD), scanning electron microscopy (SEM), transmission electron microscopy (TEM), Fourier transform infrared spectroscopy (FTIR), and the contact angle. The morphology of nanofibers and the cell adhesion capacity of DPSCs on nanofibers were evaluated using SEM. Cytocompatibility was assessed by MTT. Osteo/odontogenic differentiation ability of the nanocomposites were assessed using alkaline phosphatase (ALP) activity, alizarin red S (ARS) staining, and quantitative real-time polymerase chain reaction (qPCR) technique.

**Results:**

Viability and adhesion capacity of DPSCs were higher on P/nHAEA nanofibers than PCL and P/nHA nanofibers. ARS assay, ALP activity, and qPCR analysis findings confirmed that the nHAEA blended nanofibrous scaffolds substantially increased osteo/odontogenic differentiation of DPSCs.

**Conclusion:**

PCL/nHAEA nanocomposites had a noticeable effect on the odontogenic differentiation of DPSCs and may help to improve cell-based dentin regeneration therapies in the future.

**Supplementary Information:**

The online version contains supplementary material available at 10.1186/s12903-022-02527-1.

## Introduction

Dental pulp is a connective tissue found in the pulp chamber and root canals. The dental pulp plays a critical role in tooth regeneration following an injury by contributing in a procedure termed as dentinogenesis [1]. Dentin is the main component of the tooth and protects the dental pulp. However, dentin does not restore, and odontoblasts secrete it during their lifespan [2]. Dental caries progression can lead to pulp tissue exposure with necrosis or changes in the underlying odontoblasts. After such dentin-pulp complex deterioration, repair, or regeneration processes might occur involving newly developed hard tissue known as tertiary dentin. The only therapeutic option for promoting this regeneration process is direct pulp capping which helps develop a dentin bridge by covering the exposed pulp tissue [3, 4]. Calcium hydroxide or calcium silicate cement are now employed for direct application on exposed pulp tissue. However, the aforementioned restorative materials cannot fully repair and preserve the natural tooth system's esthetic and biomechanical properties [5]. Scientific advancements in tooth tissue engineering and stem cell technologies have demonstrated the effective practical application of dental stem cells, together with biodegradable scaffolds containing bioactive agents, for regulating dental progenitor cell differentiation, activity, and proliferation [6].

Mesenchymal stem cells (MSCs) be capable of self-renewal and multipotential differentiation. The first MSCs produced from adult human dental pulp is dental pulp stem cells (DPSCs) discovered in the pulp tissue [7]. DPSCs release a dentin matrix and have a remarkable differentiation potential to myogenic, adipogenic, angiogenic, and osteogenic cells. They can also differentiate into odontoblasts, which form dentin and bone responding to specific cytokines or growth factors [8–10]. One study revealed that DPSCs could differentiate into odontoblast-like cells capable of secreting mineralized dentin-like matrix [11]. Furthermore, Laino et al. discovered that the contact of DPSCs with the dental implant surface promoted tissue mineralization [1].

Hydroxyapatite (HA) is a biologically active mineral that is the crucial constituent of teeth and bones. It has applications in dentistry and has been used in different of scientific fields including, chemistry, biology, and medicine [12, 13]. HA is thought to be a good candidate for pulp capping. Dental pulp tissue may regenerate into complicated complex tissue-forming cells when exposed to HA. Moreover, it is integrated into the bones without toxicity and does not trigger immune responses, infection, or inflammation [14–16]. Compared to their larger micro structured counterparts, nanoscale HA has been demonstrated to enhance stem cell attachment and differentiation significantly [17]. Polycaprolactone (PCL) is an FDA-approved, nontoxic, biocompatible, and biodegradable polymer with a longer mechanical strength than other bioresorbable polymers and degrades at a rate that corresponds to bone regeneration. Adding nano hydroxyapatite (nHA) to PCL can improve the structural properties of the scaffold [18]. A composite of PCL/nHA biomaterials favors calcium phosphate mineralization followed by an osteo/odontogenic differentiation process in numerous studies [19–21].

Medicinal herbs in medical and dental procedures have a long history and have been practiced worldwide [22]. According to Iranian traditional medicine, *Elaeagnus angustifolia* (EA) is a popular herbal remedy with a variety of therapeutic effects [23] and high nutritional quality, including sodium, potassium, magnesium, calcium, iron, zinc, copper, flavonoids, and most abundant phenolic compounds [24]. Phenolic compounds raise calcium ion deposition in osteoblastic cells, enhance ALP activity, and activate osteoblast differentiation [25]. EA extract contains a lot of flavonoids, which help bone tissue by increasing osteoblastogenesis [22].

In this study, nHA was synthesized with the EA extract (nHAEA) for the first time, which could act as a reducing, stabilizing, and capping agent. Then we integrated nHAEA into PCL nanofibers (PCL/nHAEA) to stimulate odontogenic differentiation of DPSCs.

## Materials and methods

### Materials

The fruits of EA were obtained from South Khorasan, Birjand, Iran. Calcium nitrate tetrahydrate (Ca (NO_3_)_2_·4H_2_O), diammonium hydrogen phosphate ((NH_4_)_2_HPO_4_), PCL, cetylpyridinium chloride, alizarin red S, penicillin–streptomycin, MTT, Triton X-100, ascorbic acid, dexamethasone, and β-glycerol phosphate disodium salt pentahydrate were supplied from Sigma-Aldrich, St. Louis, MO. Dimethyl sulfoxide (DMSO), sodium hydroxide (NaOH) and acetic acid provided from Merck Chemical Co. Fetal bovine serum (FBS) was supplied from Capricorn, South America. Dulbecco’s modified eagle medium (DMEM) was supplied Gibco, Grand Island.

### Preparation of hydroalcoholic EA extract

Russian Olive was collected from Sout Khorasan, Birjand, Iran. A specimen was kept in the Herbarium Center of the School of Pharmacy, Birjand University of Medical Sciences. It was identified as *Elaeagnus angustifolia L*. by Dr. Sayyedeh Fatemeh Askari (Assistant Professor of Traditional Pharmacy, School of Pharmacy). Voucher number (221) was prepared for the plant in the Herbarium Center. The EA extract was made by macerating 40 g of EA powder with 320 mL methanol and 80 mL distilled water as solvents**.** Finally, the extract solution was filtered, concentrated in a rotating vacuum, and kept at 4 °C.

### Green synthesis of nHAEA

nHA and nHAEA were prepared via the sol–gel method. Calcium nitrate tetrahydrate (0.3 M, 25 mL), diammonium hydrogen phosphate (0.3 M, 15 mL), and EA extract [10% (V/V), 10 mL] was dissolved in de-ionized water. The calcium nitrate tetrahydrate solution was stirred for 0.5 h at 50 °C into the EA extract solution, and diammonium hydrogen phosphate solution was added to the complex solution drop by drop (1 mL min^−1^). To keep the pH at 11, NaOH was added to the mixture. The suspension was then stirred for 90 min at 50 °C. The resultant suspension was centrifuged and rinsed with ethanol and de-ionized water (nHAEA). Finally, an EA extract-free nHA solution was made under the same circumstances.

The stoichiometric equation for nHA is as follows:

$$30\mathrm{ Ca }{(NO3)}_{2}\cdot {4\mathrm{H}}_{2}\mathrm{O }+ 18 {({\mathrm{NH}}_{4})}_{2}{\mathrm{HPO}}_{4} + 32\mathrm{ NaOH }-\to 3 {\mathrm{Ca}}_{10}(\mathrm{PO}4{)}_{6}(\mathrm{OH}{)}_{2} + 48 {\mathrm{NH}}_{4}{\mathrm{NO}}_{3}+ 118 {\mathrm{H}}_{2}\mathrm{O }+ 32\mathrm{ Na}{\mathrm{O}}_{2}$$

FTIR and XRD were used to assess the functional groups, phase composition, and crystallinity of nHAEA and nHA. SEM and TEM were used to examine the structural and morphological properties of the produced nanoparticles.

### Electrospinning of PCL, P/nHA, and P/nHAEA nanofibrous scaffolds

PCL polymer (10% wt) was dissolved in acetic acid (90%) and stirred for 5 h in room temperature. Subsequently, nHAEA (20%wt) was added to PCL solution and stirred for 1 h. PCL/nHAEA nanofibers were synthesized by electrospinning in a 5 mL plastic syringe. The voltage between the tip of the needle and the collector on the electrospinning was 15 kV. The distance from the tip of the needle to the collector and a flow rate were 10 cm, and 1 mL/h respectively. An electrically grounded piece of aluminum foil was wrapped around the rotating drum. Finally, the solvent was evaporated and the polymer fibers assembled on the collector as nanofibers. These processes were carried out again to synthesize the PCL and PCL/nHA nanofibers. SEM, FTIR, and contact angle were used to evaluate the structural and morphological features of nanofibrous scaffolds [26, 27].

### Culture and odontogenic differentiation of DPSCs

Twenty to twenty-five years old Patients with healthy third molars were taken with consent form at the Dental Center of Hospital of Imam Reza, Birjand, Iran, with the ethics committee rules of Birjand University of Medical Sciences (ethical number: IR.BUMS.REC.1399.090). Wear prior paper discussed DPSC isolation and characterization [28]. The extracted DPSCs were cultivated in DMEM supplemented with 10% FBS and 1% pen-strep at 37 °C with 5% CO_2_ in a humidified incubator. The DPSCs from passages 3–6 were used in the subsequent analyses. To stimulate odontogenic differentiation, cells were grown in odontogenic supplement media, including DMEM supplemented with 10% FBS, 50 μM ascorbic acid, 10 mM glycerophosphate, and 10 nM dexamethasone, as discussed previously [29, 30]. During the incubation period, the culture media was replenished every 3 days.

### Cell viability assays

Cell viability assay was performed using the 3-(4–dimethylthiazol-2-yl)-2,5-diphenyl tetrazolium bromide (MTT) colorimetric test after 1, 3, and 7 days of cultivation. Before cell seeding, the nanofibers were UV sterilized for one hour. The nanofibers were placed in 96 well plates and soaked in DMEM for 24 h. Then DPSCs were seeded into each scaffold (PCL, P/nHA, and P/nHAEA) at a density of 2 × 10^4^ (cells/well). After 1, 3 and 7 days of incubation, 20 μl of MTT (2 mg/mL in PBS) add to each well and the plate incubated for 4 h in the 37 °C incubator. After converting MTT to formazan in the viable cells, which were then dissolved in 100 µL of DMSO, the formazan produced a blue-purple color. ultimately the optical absorption of the samples measured at 570 and 630 nm wavelengths using a scanning spectrophotometer (Biotek Epoch, Winooski,VT).

### Cell morphology

FE-SEM was used to assess the morphologies of DPSCs cultivated on PCL, P/nHA, and P/nHAEA scaffolds. After day 7, the cells were washed three times with phosphate-buffered saline (PBS) and were fixed in 3.5% (v/v) glutaraldehyde for 50 min at room temperature. The nanofibrous were dehydrated in a graded series of ethanol with varying ethanol concentrations (25–100%). Ultimately, all the samples were air-dried overnight for FE-SEM analysis (Tescan MIRA3).

### Alizarin red S assay

DPSCs were seeded at a density of 10^5^ cells/scaffold and were incubated for 14 days in odontogenic differentiation media. On day 14, the cell scaffolds were rinsed twice with PBS, fixed for 1 h at room temperature in ice-cold 70% ethanol, washed in distilled water, and stained with ARS dye (40 mM, pH 4.2) for 30 min at ambient temperature. To remove the background, scaffolds with no cells (PCL, P/nHA, and P/nHAEA) were applied. After washing the materials in de-ionized water and incubating them with 10% cetylpyridinium chloride to calcium ions desorption. the absorbance of the solution was determined at 570 nm.

### Alkaline phosphatase activity

DPSCs were seeded at a density of 10^5^ cells/scaffold and were incubated for 14 days in odontogenic differentiation media. The DPSC-seeded nanofibers were retrieved from the culture plates after 14 days and washed with PBS before being dissolved with 0.1% Triton X-100. After that, the samples were sonicated for 5 min and the mixture was centrifuged at 12,000 g for 20 min at 4 °C and the supernatant was collected. The ALP assay Kit (Biorexfars, Iran) was used to measure ALP activity in cell supernatants according to protocol of manufacturer. An ELISA reader was used to measure absorbance at 405 nm.

### Quantitative real-time polymerase chain reaction (qPCR)

PCL, P/nHA, and P/nHAEA scaffolds were rinsed with PBS after 21 days of odontogenic differentiation induction. RNA was extracted using the total RNA Extraction Kit (Pars Tous, Tehran, Iran). The quality of samples was measured at 260/280 nm with a nano Drop (Biotek Epoch). 2 μg of total RNA was then used to synthesize cDNA (Pars Tous). The Oligo 7 primer tool was used to design specific primers for BMP2, Runx2, and DSPP genes (Table 1). The qPCR analysis was carried out using SYBR Green PCR Master Mix (Amplicon, Denmark) in a total volume of 25 µL. The ABI Step One Plus real-time PCR machine (Applied Biosystems) was used to conduct the cDNA amplification. The thermal cycling parameters were denaturation step at 95 °C for 10 min followed by 40 cycles at 95 °C for 30 s, 60 °C for 30 s, and 72 °C for 30 s. A melting curve was established after an additional step ranging from 60 to 95 °C. This was utilized to test the specificity of each primer pair's qPCR reaction. The 2^−ΔΔCt^ technique was used to normalize target gene expression against GAPDH as an endogenous control in the study.

**Table 1:** Sequences of primers used for quantitative real-time polymerase chain reaction (qPCR)

Name	Forward	Reverse	Product (bp)
BMP2	GAGAAGGAGGAGGCAAAGAAAAG	GAAGCAGCAACGCTAGAAGAC	183
Runx2	ACCTTGACCATAACCGTCTTC	GGCGGTCAGAGAACAAACTA	145
DSPP	TTCCGATGGGAGTCCTAGTG	TCTTCTTTCCCATGGTCCTG	144
GAPDH	CGAACCTCTCTGCTCCTCCTGTTCG	CATGGTGTCTGAGCGATGTGG	83

### Statistical analysis

All experiments were carried out in triplicate, and GraphPad Prism software, Version 9 (GraphPad Software, Inc, La Jolla, CA) was used for statistical analysis. Mean, and standard deviation (SD) were determined (Additional file). A one-way analysis of variance (ANOVA) was used to conduct mean comparison studies followed by a Tukey's multiple comparisons test, with *p* < 0.05 considered significant. Significant differences between groups are indicated in histograms by the following asterisks; *p < 0.05; **p < 0.01; and ***p < 0.001.

## Result

### XRD analyses of nanoparticles

**Fig. 1:**
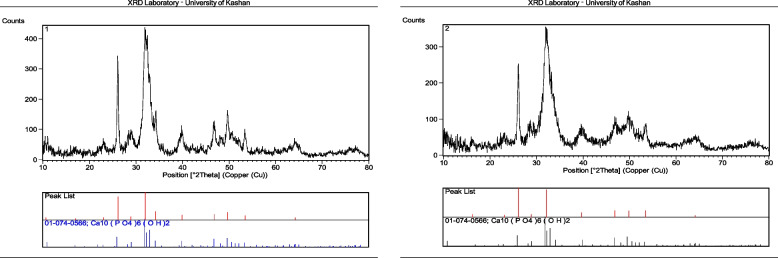
XRD patterns of nHA, and nHAEA

The X-ray diffraction patterns of the produced nanoparticles are shown in Fig. 1, nHAEA, and nHA having the same XRD pattern. The lack of calcium hydroxide and calcium phosphate peaks demonstrated that the nanomaterials were pure. The peaks detected at 26◦, 32◦, 39◦, 46◦, 49◦,53◦ and 64◦, were compatible with the planes (002), (112), (310), (222), (123), (004), and (233), respectively, showing the presence of HA, which corresponded to ICDD: 01–084-1998.

### Morphology of nanoparticles

**Fig. 2:**
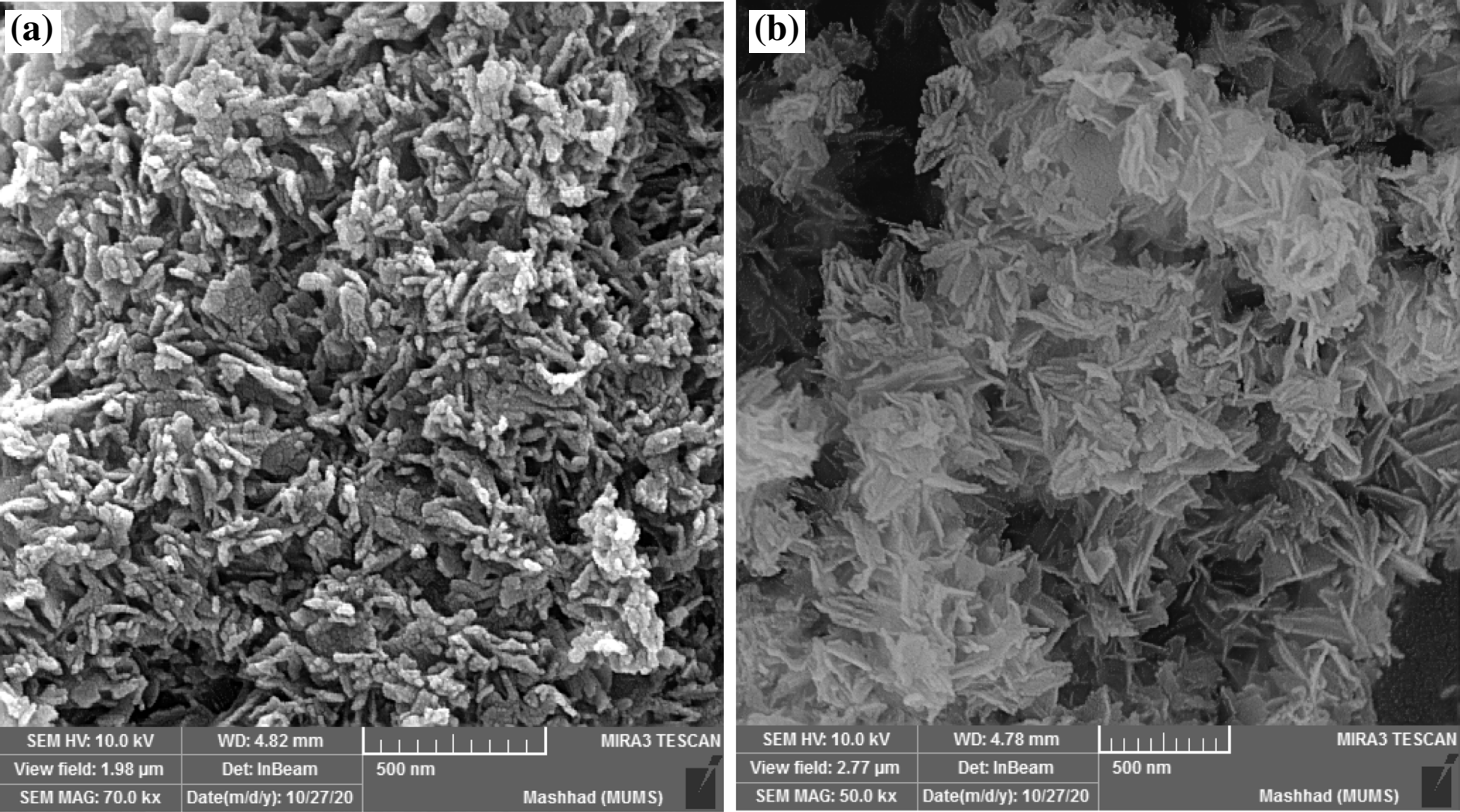
FESEM images of (**a**) nHA, and (**b**) nHAEA

**Fig. 3:**
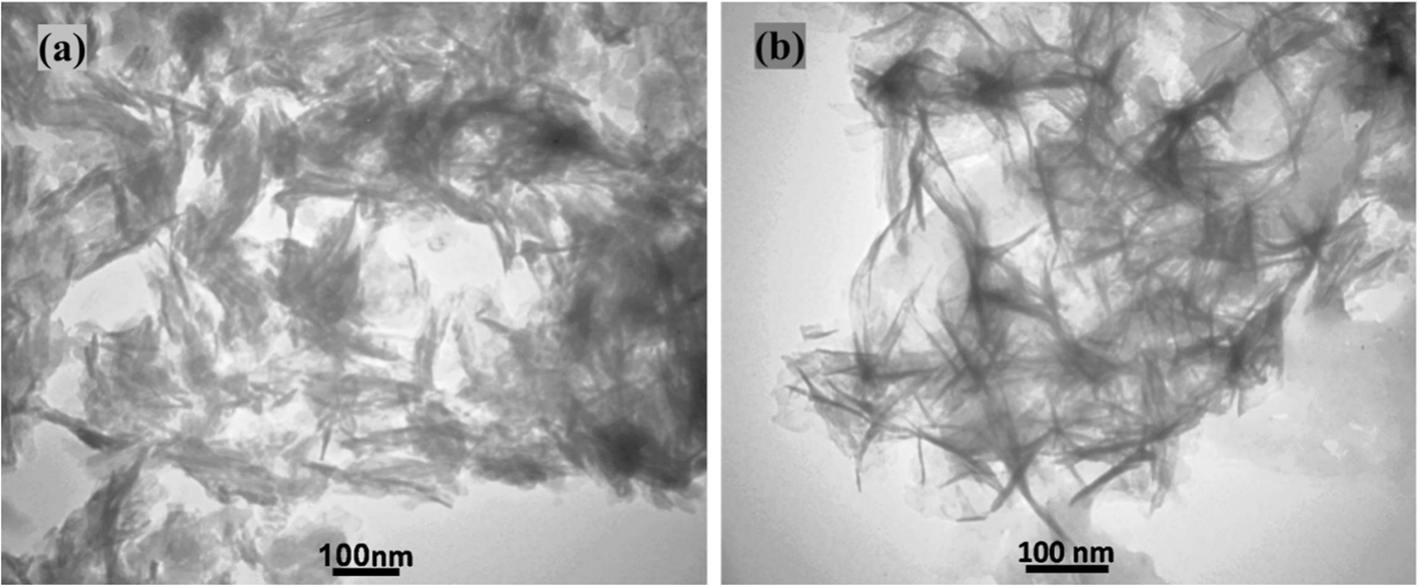
TEM images of (**a**) nHA, and (**b**) nHAEA

According to the study's analysis of FESEM and TEM images, the particles were nanorods (Figs. 2 and 3). The nHAs had a width of 17–29 nm and a length of 62–89 nm, whereas nHAEA nanorods had a width of 17–23 nm and a length of 93–146 nm.

### FTIR

**Fig. 4:**
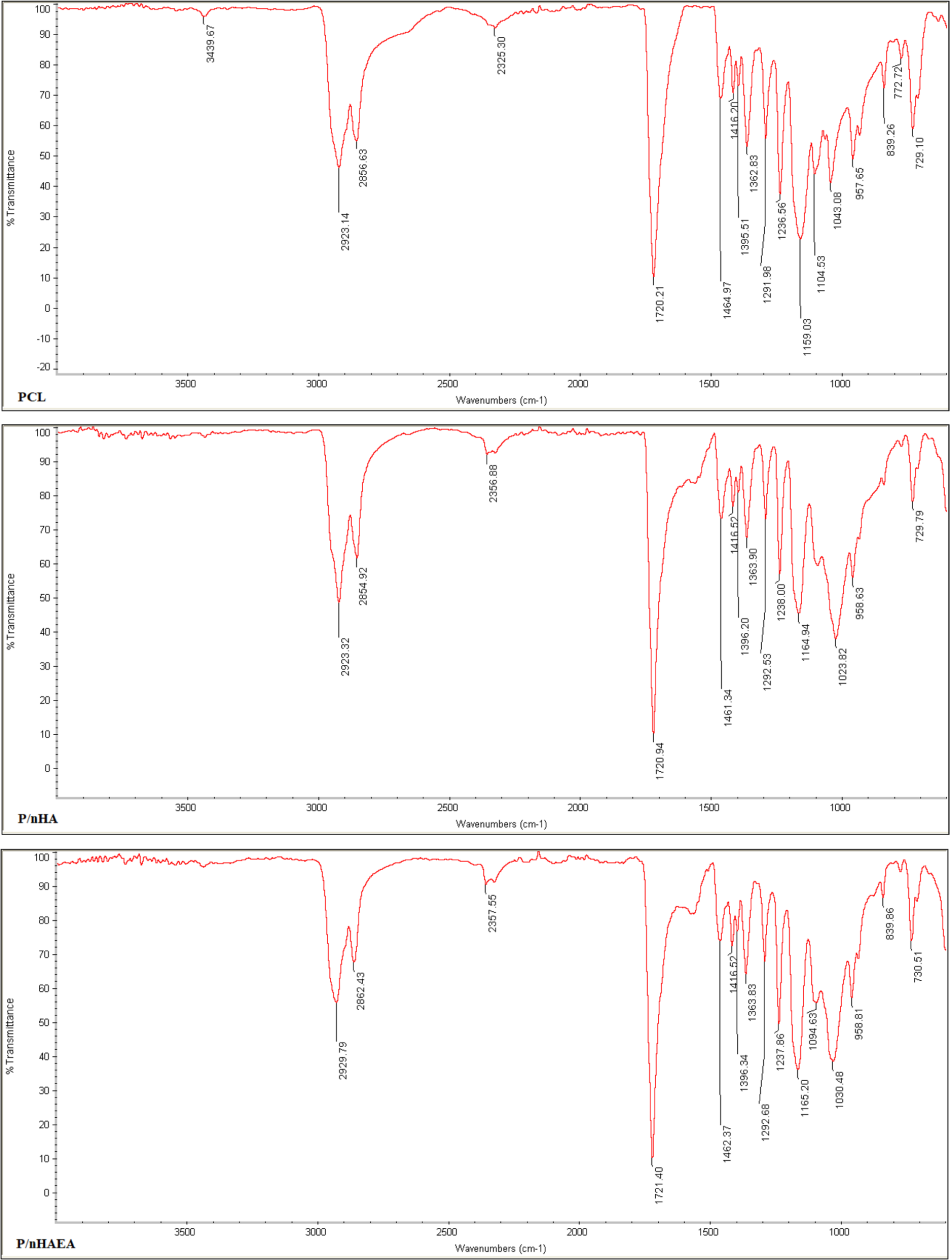
FTIR spectra of PCL, P/nHA, and P/nHAEA nanofibers

As shown in Fig. 4, the functional groups of PCL, P/nHA, and P/nHAEA were characterized using FTIR in the 400–4000 cm^−1^ range. The distinct bands of 1023 and 1030 in the P/nHA and P/nHAEA, respectively were indicated to be PO4^3−^. Several distinct PCL peaks were detected for three different types of scaffolds at 1720 cm^−1^, 1292 cm^−1^, around 1240 cm^−1^ which could be attributed to (C = O stretching), (C–O and C–C stretching), (C–O–C asymmetric stretching) respectively. Also, three peaks at 1363, around 2854, and 2923 cm^−1^ could be assigned to C–H in PCL. All of the characteristic peaks for PCL and nHAs indicated that nHA and nHAEA were successfully incorporated into PCL.

### Morphology of nanofibrous scaffolds

**Fig. 5:**
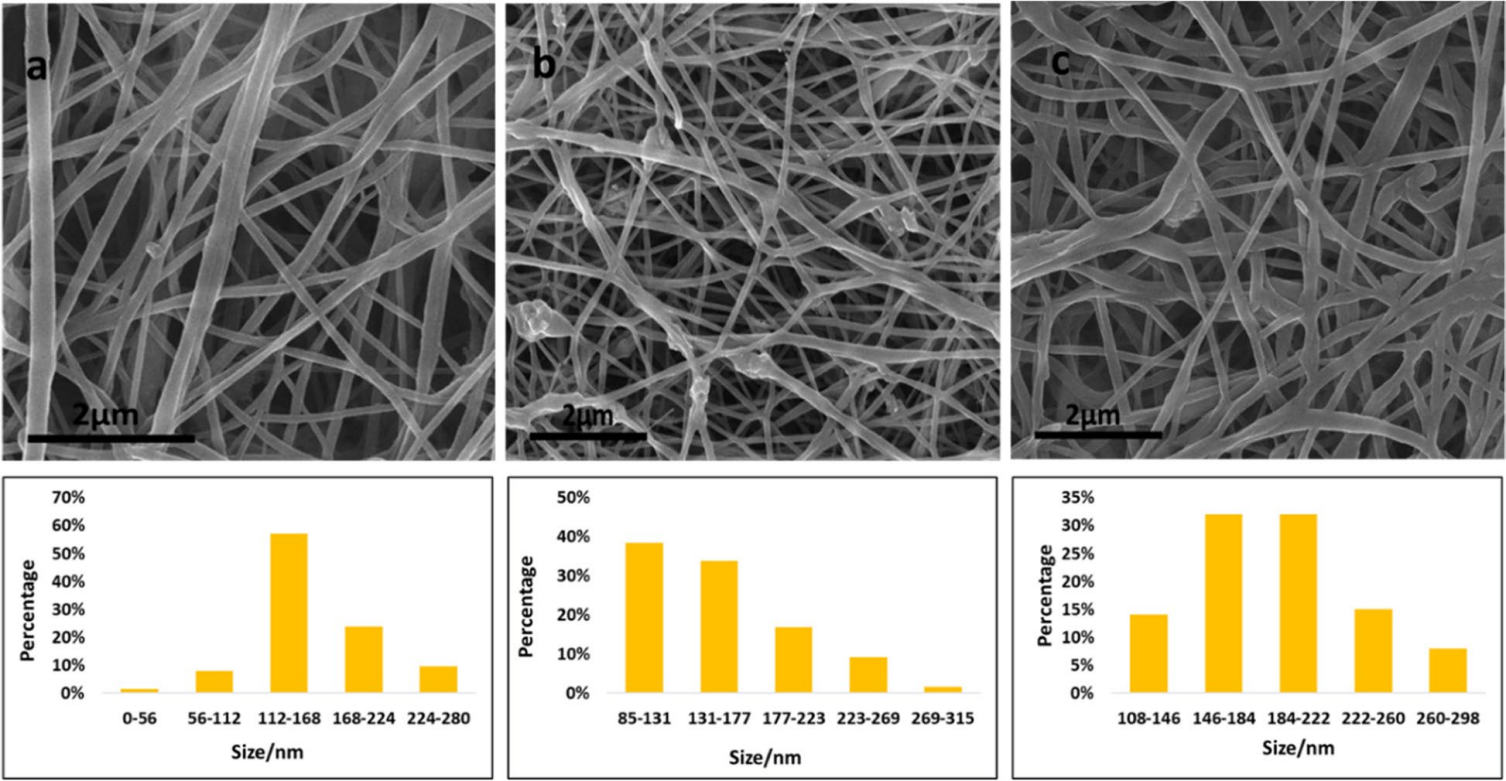
FESEM micrographs of (**a**) PCL, (**b**) P/nHA, and (**c**) P/nHAEA nanofibers and corresponding fiber diameter distributions. Scale bar: 2 µm

FESEM was used to examine the morphology and structure properties of the nanofibers (Fig. 5). The PCL nanofibrous average diameter was 164.9 ± 41.3 nm, while the P/nHA, and P/nHAEA nanofibrous were 156.8 ± 49.4 and 191.8 ± 43.1 nm, respectively.

### Hydrophilicity of the nanofibrous scaffolds

The contact angle measurement was carried out in order to comprehend the impact of the surface wetting potential of nanofibrous scaffolds. The prepared scaffold's water contact angle data are shown in the Table 2. The incorporation of nHA nanoparticles into the PCL nanofibrous scaffold enhanced the hydrophobicity of its surface and resulted in the increased contact angle value (107°) compare than PCL (96°). The contact angle of the P/nHAEA nanofibers was 85°.

**Table 2:**
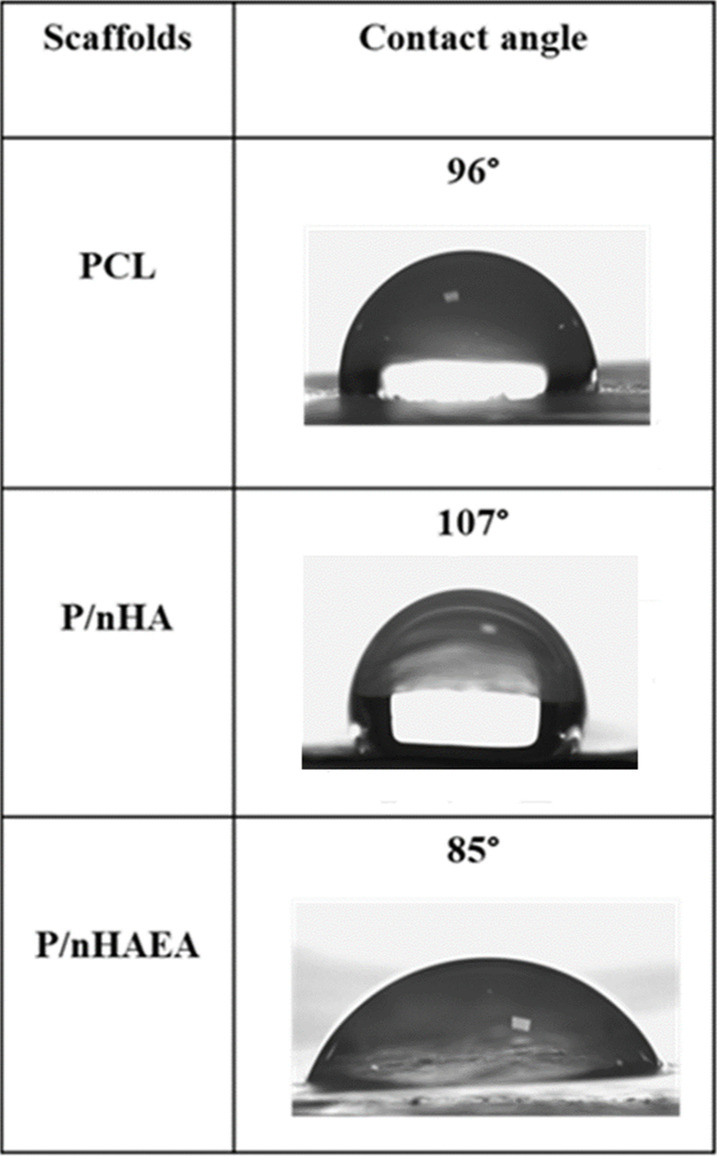
Water contact angles of PCL, P/nHA, and P/nHAEA nanofibrous scaffolds

### MTT assay

**Fig. 6:**
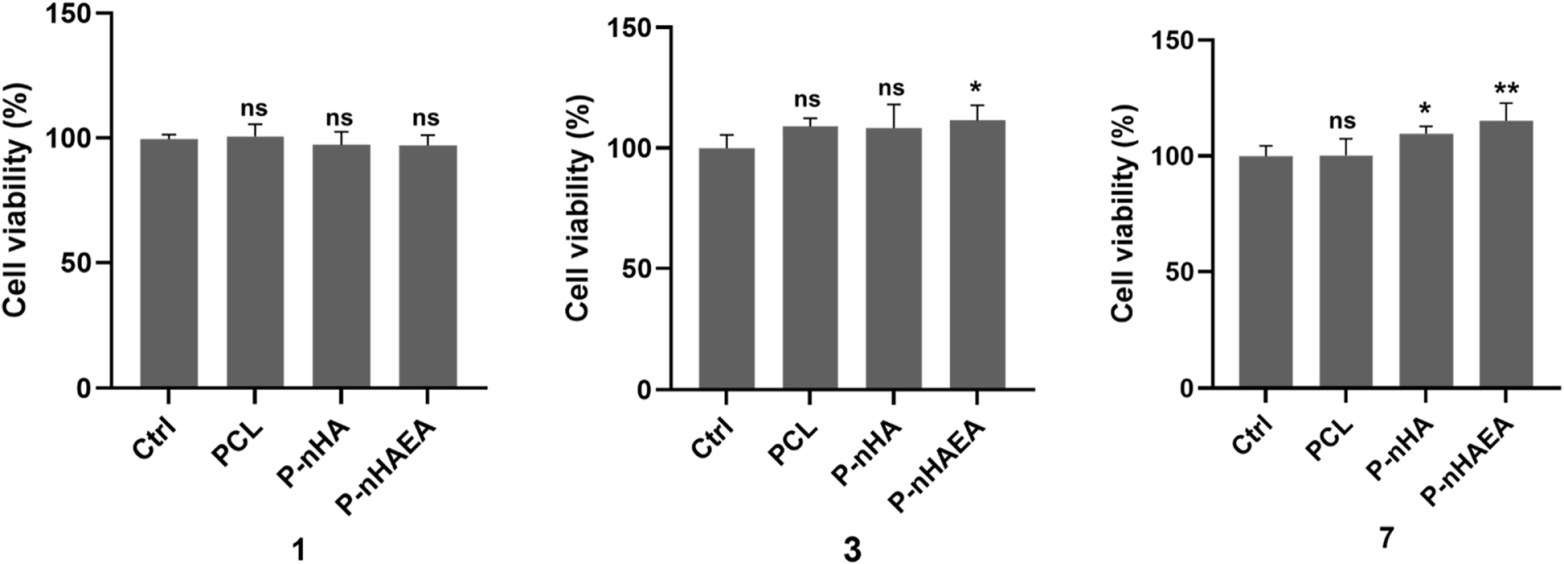
MTT assay results for DPSCs cultured on ctrl, PCL, P/nHA, and P/nHAEA nanofibers for 1, 3, and 7 days. Significant differences compared to control were indicated: *p < 0.05; **p < 0.01; ***p < 0.001; ctrl, Control (without scaffold); ns, nonsignificant

MTT assay was carried out to investigate the viability of DPSCs on nanofibrous scaffolds. Figure 6 depicts the MTT absorbance values of DPSCs seeded on PCL, P/nHA, and P/nHAEA after 1, 3, and 7 days of cell culture. After one day, there was no significant difference between the different groups. The viability of cells significantly increased for P/nHAEA on day 3 (*p* < 0.05) and especially for P/nHA (*p* < 0.05) and P/nHAEA(*p* < 0.01) on day 7. These findings revealed that nHAEAs are nontoxic and have a favorable cytocompatibility with DPSCs.

### The adhesion of DPSCs on the nanofibrous scaffolds

**Fig. 7:**
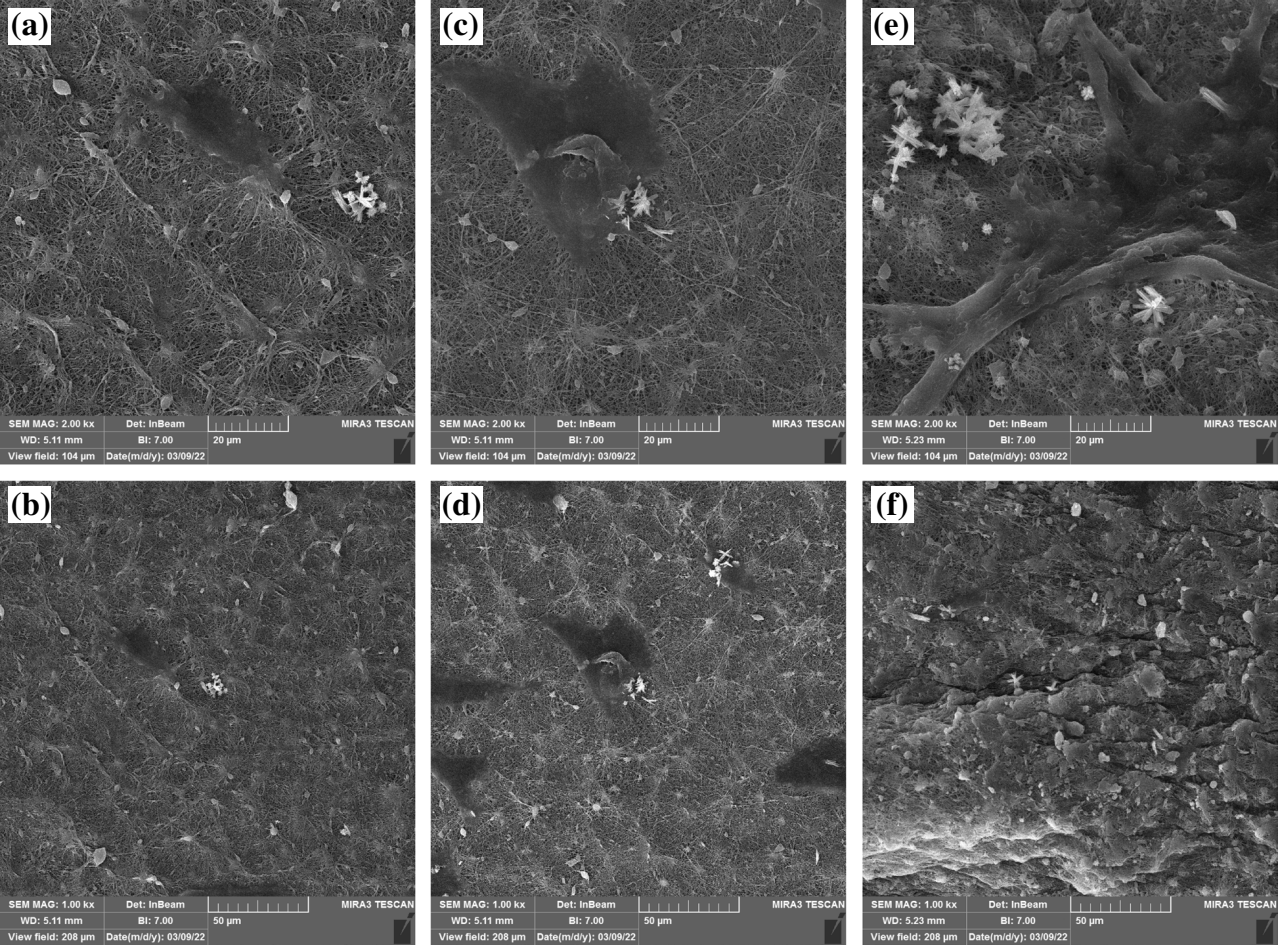
FESEM images of DPSCs that were adhered on (**a**, **b**) PCL, (**c**, **d**) P/nHA, (**e**, **f**) P/nHAEA electrospun nanocomposites after 7 days of culture. Scale bars: (**a**, **c**, **e**) = 20 µm, (**b**, **d**, **f**) = 50 µm

DPSCs morphology on the PCL, P/nHA, and P/nHAEA nanofibers scaffolds are shown in Fig. 7. FESEM images showed an enlarged and spindle fibroblast-like shaped morphology on the PCL, P/nHA, and P/nHAEA nanofibers scaffolds after 7 days of culture. The DPSCs on the nanofiber scaffolds were capable of spreading, demonstrating that the DPSCs and nanofibers have close contacts. The P/nHAEA nanofibrous scaffolds had better attachment and distribution of DPSCs (Figs. 7e, f) than the PCL and P/nHA nanofibrous scaffolds (Figs. 7a, b, c, d).

### Mineralization of DPSCs on the nanofibrous scaffolds

**Fig. 8:**
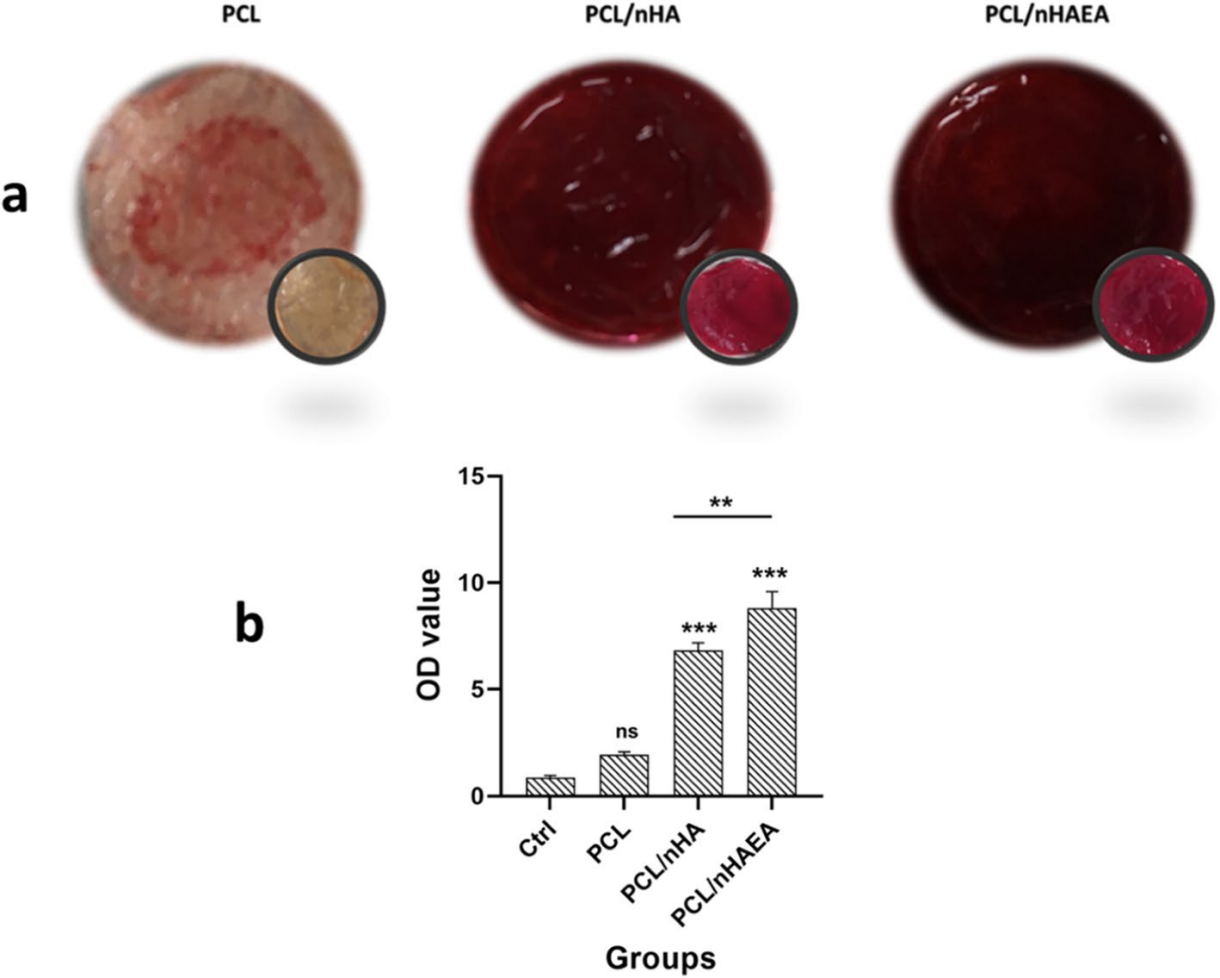
Effect of PCL, P/nHA and, P/nHAEA nanofibrous scaffolds on DPSCs osteogenic differentiation: (**a**) Representative images of scaffolds stained with alizarin red dye at the 14 days. Scaffolds without cells stained with alizarin red for background removal (blank) (**b**) A bar chart demonstrating quantitative results of differentiated DPSCs stained with alizarin red S after 14 days of culture. *p < 0.05, **p < 0.01, ***p < 0.001 for each group compared to the control group;  , differences between groups; ctrl, Control (without scaffold); ns, nonsignificant

ARS assay was used to visually detect of mineralization of DPSCs on PCL, P/nHA, and P/nHAEA nanofibers after 14 days of culture (Fig. 8). The calcium deposition on the P/nHA and P/nHAEA composite scaffolds was significantly greater than on the PCL and control (*p* < 0.001). Moreover, quantitative findings confirmed that cells treated with P/nHAEA had considerably greater alizarin red activity than cells treated with P/nHA (*p* < 0.01).

### Alkaline phosphatase activity of DPSCs on the nanofibrous scaffolds

**Fig. 9:**
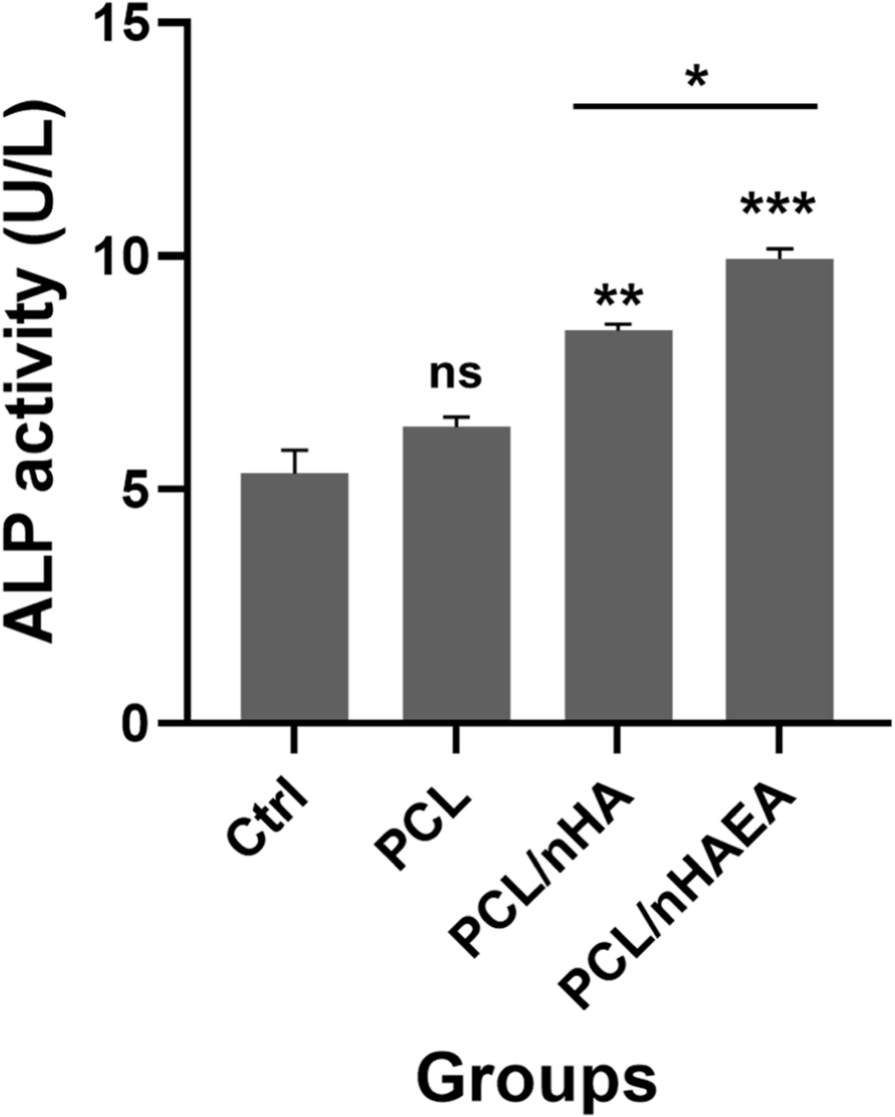
ALP activity of DPSCs cultured on P/nHA, and P/nHAEA nanofibrous scaffolds. *p < 0.05, **p < 0.01, ***p < 0.001 for each group compared to the control group;  , differences between groups; ctrl, Control (without scaffold); ns, nonsignificant

Figure 9 depicts the ALP activity of DPSCs seeded on PCL, P/nHA, and P/nHAEA after 14 days. The ALP activity of P/nHA (*p* < 0.01) and P/nHAEA (*p* < 0.001) was noticeably greater than that of the PCL and control groups. The ALP activity of the PCL was not significantly different with the control group.

### Quantitative real-time polymerase chain reaction (qPCR)

**Fig. 10:**
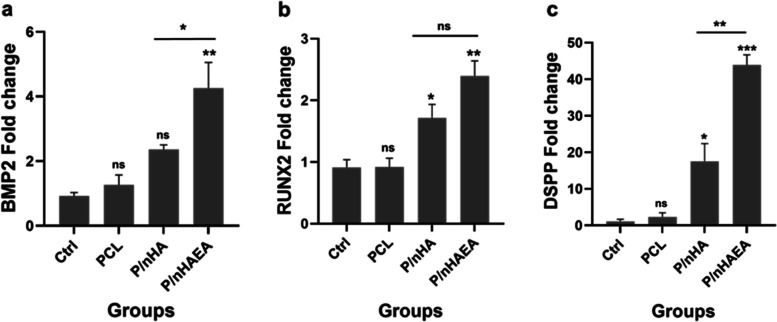
The expression of (**a**) BMP2, (**b**) Runx2, and (**c**) DSPP genes in DPSCs seeded in three different scaffolds PCL, P/nHA, P/nHAEA, and control (without scaffold). *p < 0.05, **p < 0.01, ***p < 0.001 for each group compared to the control group;  , differences between groups; ctrl, Control (without scaffold); ns, nonsignificant

The expression levels of BMP-2, Runx2, and DSPP were measured to determine the influence of nHA, and nHAEA integrated PCL on odontogenic differentiation of DPSCs (Fig. 10). The BMP-2 gene expression in PCL and P/nHA group had no significant effect compared to the control group, but it was substantially higher in P/nHAEA than in the other groups (*p* < 0.01). For Runx2, P/nHAEA exhibited the most significant increase in expression (*p* < 0.01), and P/nHA also had a significant effect (*p* < 0.05), but the PCL group had no significant practical impact compared to the control group. Similarly, DSPP gene expression was enhanced in P/nHAEA (*p* < 0.001) and P/nHA (*p* < 0.05). These findings imply that nHAEA can improve the osteoconductivity of PCL nanofibers.

## Discussion

The tissue engineering method offers a novel approach to dentin restoration by regenerating dentin to replace damaged tooth tissue. DPSCs are used for dental tissue engineering and regeneration in combination with scaffolds because of their excellent availability, great accessibility in the oral cavity, their ability to differentiate into odontogenic cells, and their potential for dentin regeneration [1, 31, 32]. Because dentin and bone structures are so similar, investigations in dental tissue engineering are frequently conducted in association with bone formation processes and use osteoinductive materials [33]. nHAs have been widely and successfully used for osteo/odontogenic regeneration [17, 34, 35]. Plant extract-assisted green synthesis has gotten much interest and has become a hot topic in nanotechnology research [36]. EA extract may induce osteogenic differentiation of the DPSC. As a result, the current study explores the impact of this new composite based on PCL and nHA modified with EA extract, the prevalent polyphenolic compounds, on enhancing the osteo/odontogenic differentiation of DPSCs for the first time. For this purpose, PCL, P/nHA, and P/nHAEA scaffolds were manufactured for odonto/osteogenic differentiation of DPSCs.

The XRD patterns of nanoparticles verified that nHAEA and nHA have the same XRD patterns without any additional phosphate phases. The SEM and TEM images of nanoparticles revealed ellipsoidal and rod-like forms in the nanoscale. The FTIR spectroscopic analysis confirmed the functional groups of the PCL as well as the incorporation of nHA and nHAEA in the composites. According to SEM images result of nanocomposites, Surface morphology of PCL nanofibers were similar to homogenous fibers. The incorporated nHA and nHAEA created a little change in nanofibers matrix morphology. In the case of P/nHA nanofibers, some aggregated nHA particles were observed. nHA penetrates via the fibers walls at some places, increasing their surface roughness [27]. However, the nHAEA was well-dispersed throughout the fiber, with minor aggregation.

The PCL nanofibrous average diameter was 164.9 ± 41.3 nm, while the P/nHA and P/nHAEA nanofibrous were 156.8 ± 49.4 and 191.8 ± 43.1 nm, respectively. It was discovered that adding nHA into the PCL mixture could decrease the fiber diameter. The incorporation of nHA into the PCL mixture appears to have the potential to boost net charge density during electrospinning. An augment in net charge should enhance the electrostatic force inside the jet and result in more plane stretching. As a result, a decline in fiber diameter [19]. Since the viscosity of the spinning mixtures effects the thickness of nanocomposites, and the presence of EA increased the viscosity of the polymer solution, the P/nHAEA solutions produced fibers with a greater diameter [22].

Water contact angle measurements were used to assess the surface hydrophilicity of the nanofibers. PCL nanofibers had a contact angle of 96°, while P/nHA and P/nHAEA nanofibers had contact angles of 107° and 85°, respectively. According to Rajzer's research, integrating nHA into the PCL nanocomposites scaffold enhanced the hydrophobicity of its surface and resulted in the highest contact angle value [19]. The EA plant extract, which contains various hydrophilic components such as polysaccharides, proteins, and other hydrophilic components, may be responsible for the increase in hydrophilicity in the P/nHAEA group [22].

Cell contact, attachment, and spreading have been reported as the first phase in cell-material interactions, which influence cell proliferation and differentiation capacity [32]. The current study found that the P/nHAEA group had improved intercellular and cell scaffold adherence capabilities, implying that EA extract plays a significant role in DPSC proliferation and differentiation.

In our study, the alizarin red staining experiment indicated that DPSCs successfully differentiated into odontogenic cells. The cells grown on P/nHAEA nanocomposite scaffolds had more significant levels of deposited calcium than PCL and P/nHA.

ALP activity is one of the most crucial markers of odontoblasts, since differentiated odontoblasts have substantially higher ALP activity than dental undifferentiated mesenchymal cells. The activity of this enzyme is required for pulp cells to develop into odontoblasts [37]. A considerable boost in ALP activity of cells grown with P/nHAEA was reported in this investigation for 14 days.

According to our study, the viability of cells cultured onto nanofibers after 1, 3, and 7 days indicates that the nanofiber mats have no cytotoxicity and showed in vitro biocompatibility. In addition, the cell viability of P/nHAEA increased with increasing cell culture time. Soares et al. revealed that nHA-embedded PCL nanofibers scaffolds are cytocompatible and can enhance human dental pulp cells' adherence and odontogenic capacity [5].

Our finding showed that the expression levels of the marker genes for odontogenic differentiation (BMP2, Runx2, and DSPP) were significantly higher in the P/nHAEA than in the P/nHA, confirming that the incorporation of nHAEA into the PCL enhanced the osteo/odontogenic differentiation of the DPSCs. Runx2 is a critical factor in odontoblastic differentiation. Furthermore, it is a key transcription factor in osteoblastic differentiation and bone formation [17, 38]. BMP2 is a non-collagenous protein found in the dentin matrix that regulates odontogenic differentiation and mineralization during dentinogenesis [7]. DSPP is also a specific marker for odontogenic differentiation and is crucial for dentinogenesis and regeneration of the dentin-pulp complex [39–41].

Prior studies planned for this investigation and the results for BMP2, DSPP, and Runx2 overexpression are consistent with other designs that have been employed to improve osteoconductivity [7, 22, 34]. Chuenjitkuntaworn et al. demonstrated that three types of mesenchymal stem cells could grow on a PCL/HA scaffold. Furthermore, when compared to other groups, DPSCs with PCL/HA had the higher level of calcium deposition and were more compatible with these scaffolds than BMSCs and ADSCs [21]. Hokmabad et al*.* proved that loading EA extracts with PCL-PEG-PCL nanofibrous scaffolds promoted DPSC survival, proliferation, and osteogenic differentiation by enhancing ALP activity and calcium deposition, and the expression of BMP2, DSPP, BGLAP, and Runx2 [22]. Also, Asghari et al. discovered that PCL/PLA/HA nanofibrous scaffolds could induce odontogenic and osteogenic differentiation of DPSCs by expressing BMP2, DSPP, Osteocalcin, and Runx2 [34].

## Conclusion

According to the findings of this investigation, combining modified nHA via EA extract with PCL had a noticeable favorable influence on the odontogenic differentiation of DPSCs. PCL/nHAEA increased DPSC viability, attachment, and differentiation compared to other groups. Also, the expression of odonto/osteogenic genes in DPSCs were upregulated. Our findings will be the basis for the future use of PCL/nHAEA nanocomposite for cell-based dentin regeneration therapies. Although these results are promising, further in vivo studies to investigate the role of the PCL/nHAEA nanocomposite on reparative dentin formation and dental pulp vitality preservation are required.

## Supplementary Information

**Additional file 1:** **Table 1.** Atomicpercentage of nanorods. nHAEA; modified nanohydroxyapatite via EA extract nHA; nanohydroxyapatite. **Table 2.** Mean and standard deviation (SD) values of cell viability inMTT assay. Ctrl; Control, DMEM, PCL; Polycaprolactone,P/nHA; Polycaprolactone/nanohydroxyapatite, P/nHAEA; Polycaprolactone/ modified nanohydroxyapatite viaEA extract. **Table 3.** Mean and standarddeviation (SD) values of relative gene expression in q-PCR assessment. **Table4.** Mean and standard deviation (SD)values of ALP activity assessment. **Table 5. **Mean and standard deviation (SD) values of ARS assessment. 

## Data Availability

All data obtained or assessed during this investigation are contained in this article as "Additional file " in the Statistical analysis part of the method portion.

## Abbreviations

DPSC: Dental pulp stem cell
EA: Elaeagnus angustifolia
PCL: Polycaprolactone
PCL/nHAEA: Polycaprolactone/ modified nanohydroxyapatite via EA extract
P/nHAEA: Polycaprolactone/ modified nanohydroxyapatite via EA extract
P/nHA: Polycaprolactone/nanohydroxyapatite
BMP2: Bone morphogenetic protein 2
Runx2: Runt-related transcription factor 2
DSPP: Dentin sialophosphoprotein
ALP: Alkaline phosphatase
