# Supplementary material for: Effect of PCL/nHAEA nanocomposite to osteo/odontogenic differentiation of dental pulp stem cells
Source: BMC Oral Health. 2022 Nov 16;22:505. doi: 10.1186/s12903-022-02527-1 (PMC9670388; doi:10.1186/s12903-022-02527-1)
Supplement: Supplementary file 1 — Additional file 1: Table 1. Atomicpercentage of nanorods. nHAEA; modified nanohydroxyapatite via EA extract nHA; nanohydroxyapatite. Table 2. Mean and standard deviation (SD) values of cell viability inMTT assay. Ctrl; Control, DMEM, PCL; Polycaprolactone,P/nHA; Polycaprolactone/nanohydroxyapatite, P/nHAEA; Polycaprolactone/ modified nanohydroxyapatite viaEA extract. Table 3. Mean and standarddeviation (SD) values of relative gene expression in q-PCR assessment. Table4. Mean and standard deviation (SD)values of ALP activity assessment. Table 5. Mean and standard deviation (SD) values of ARS assessment. [file 12903_2022_2527_MOESM1_ESM.docx]

Table 1. Atomic percentage of nanorods. nHAEA; modified nanohydroxyapatite via EA extract, nHA; nanohydroxyapatite

| **Types of nanorods** | **Ca (at. %)** | **P (at. %)** | **O (at. %)** | **Ca/P (at. %)** |
| --- | --- | --- | --- | --- |
| **nHA** | **19.59** | **12.73** | **67.68** | **1.54** |
| **nHAEA** | **21.45** | **14.99** | **63.56** | **1.43** |

Table 2. Mean and standard deviation (SD) values of cell viability in MTT assay. Ctrl; Control, DMEM, PCL; Polycaprolactone, P/nHA; Polycaprolactone/nanohydroxyapatite, P/nHAEA; Polycaprolactone/ modified nanohydroxyapatite via EA extract

| **SD** | **Mean** | **Groups** | **Time** |
| --- | --- | --- | --- |
| **1.834** | **99.53** | **Ctrl** | **1 day** |
| **4.928** | **100.5** | **PCL** |  |
| **5.270** | **97.23** | **P-nHA** |  |
| **3.953** | **97.12** | **P-nHAEA** |  |
| **5.429** | **100.0** | **Ctrl** | **3 days** |
| **3.402** | **108.9** | **PCL** |  |
| **9.793** | **108.3** | **P-nHA** |  |
| **6.232** | **111.4** | **P-nHAEA** |  |
| **4.398** | **100.0** | **Ctrl** | **7 days** |
| **7.187** | **100.3** | **PCL** |  |
| **3.122** | **109.7** | **P-nHA** |  |
| **7.613** | **115.3** | **P-nHAEA** |  |

Table 3. Mean and standard deviation (SD) values of relative gene expression in q-PCR assessment.

| **SD** | **Mean** | **Groups** | **Targeted gene** |
| --- | --- | --- | --- |
| **0.1018** | **0.9280** | **Ctrl** | **BMP2** |
| **0.3111** | **1.260** | **PCL** |  |
| **0.1414** | **2.360** | **P-nHA** |  |
| **0.7920** | **4.260** | **P-nHAEA** |  |
| **0.1287** | **0.9090** | **Ctrl** | **Runx2** |
| **0.1442** | **0.9180** | **PCL** |  |
| **0.2192** | **1.715** | **P-nHA** |  |
| **0.2475** | **2.395** | **P-nHAEA** |  |
| **0.04950** | **1.000** | **Ctrl** | **DSPP** |
| **1.103** | **2.350** | **PCL** |  |
| **4.844** | **17.55** | **P-nHA** |  |
| **2.793** | **43.91** | **P-nHAEA** |  |

Table 4. Mean and standard deviation (SD) values of ALP activity assessment.

| **SD** | **Mean** | **Groups** | **Targeted gene** |
| --- | --- | --- | --- |
| **0.4907** | **5.347** | **Ctrl** | **ALP activity** |
| **0.2121** | **6.350** | **PCL** |  |
| **0.1414** | **8.400** | **P-nHA** |  |
| **0.2121** | **9.950** | **P-nHAEA** |  |

Table 5. Mean and standard deviation (SD) values of ARS assessment.

| **SD** | **Mean** | **Groups** | **Targeted gene** |
| --- | --- | --- | --- |
| **0.1051** | **0.8663** | **Ctrl** | **ARS** |
| **0.1481** | **1.927** | **PCL** |  |
| **0.3477** | **6.833** | **P-nHA** |  |
| **0.7731** | **8.812** | **P-nHAEA** |  |
